# Supplementary material for: Enhancing the Therapeutic Effect of 2-211At-astato-α-methyl-L-phenylalanine with Probenecid Loading
Source: Cancers (Basel). 2021 Nov 3;13(21):5514. doi: 10.3390/cancers13215514 (PMC8583516; doi:10.3390/cancers13215514)
Supplement: Supplementary file 1 [file cancers-13-05514-s001.zip › cancers-1452784-supplementary.pptx]

## Slide 1
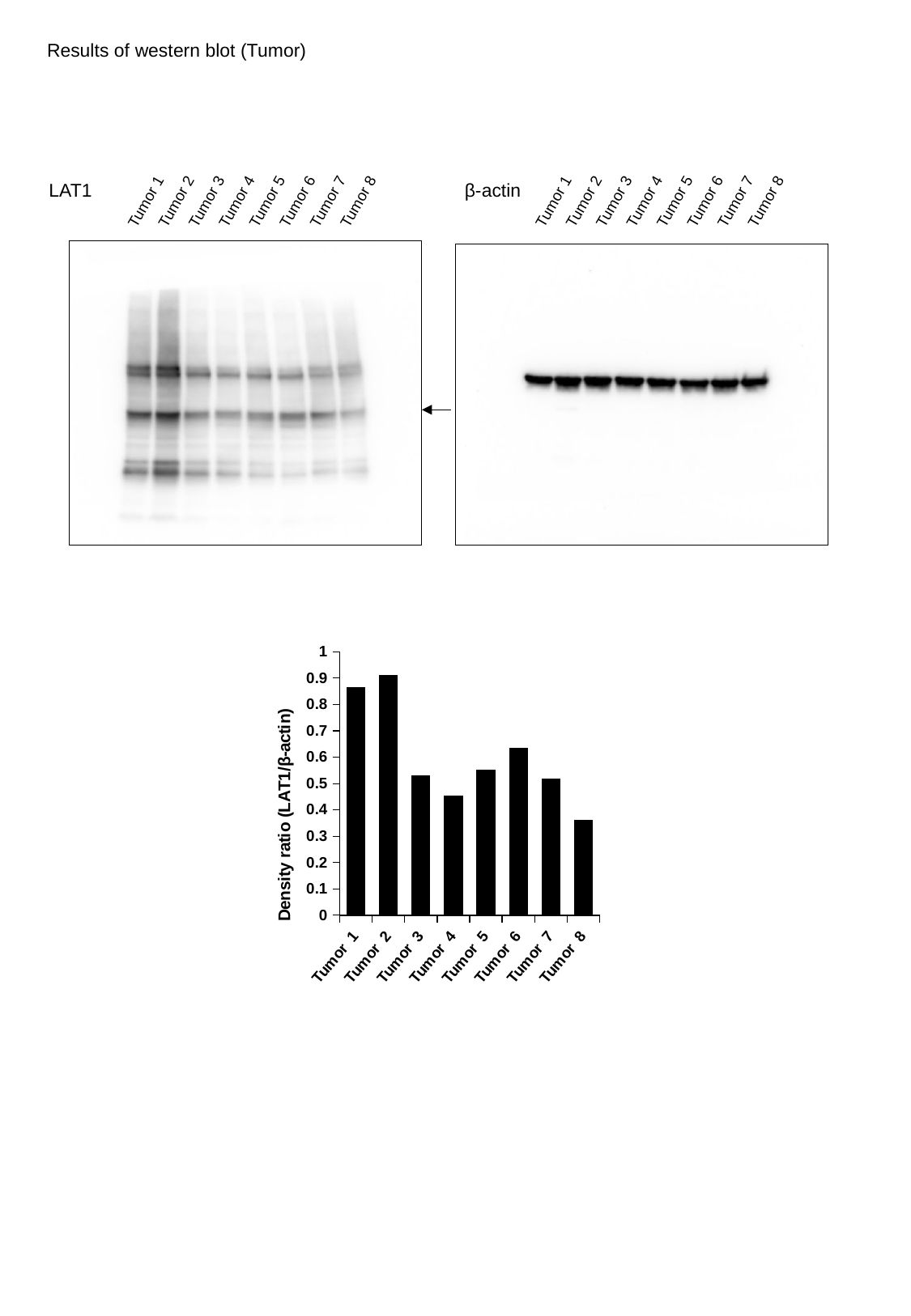

Results of western blot (Tumor)
LAT1
β-actin
Tumor 2
Tumor 1
Tumor 3
Tumor 4
Tumor 5
Tumor 6
Tumor 7
Tumor 8
Tumor 2
Tumor 1
Tumor 3
Tumor 4
Tumor 5
Tumor 6
Tumor 7
Tumor 8
### Chart
| Category | |
|---|---|
| Tumor 1 | 0.86466475154806 |
| Tumor 2 | 0.9103576780797706 |
| Tumor 3 | 0.5302012770502723 |
| Tumor 4 | 0.4543721338518858 |
| Tumor 5 | 0.5499780259151144 |
| Tumor 6 | 0.6336909055342801 |
| Tumor 7 | 0.5180202726611806 |
| Tumor 8 | 0.3602910408564245 |

## Slide 2
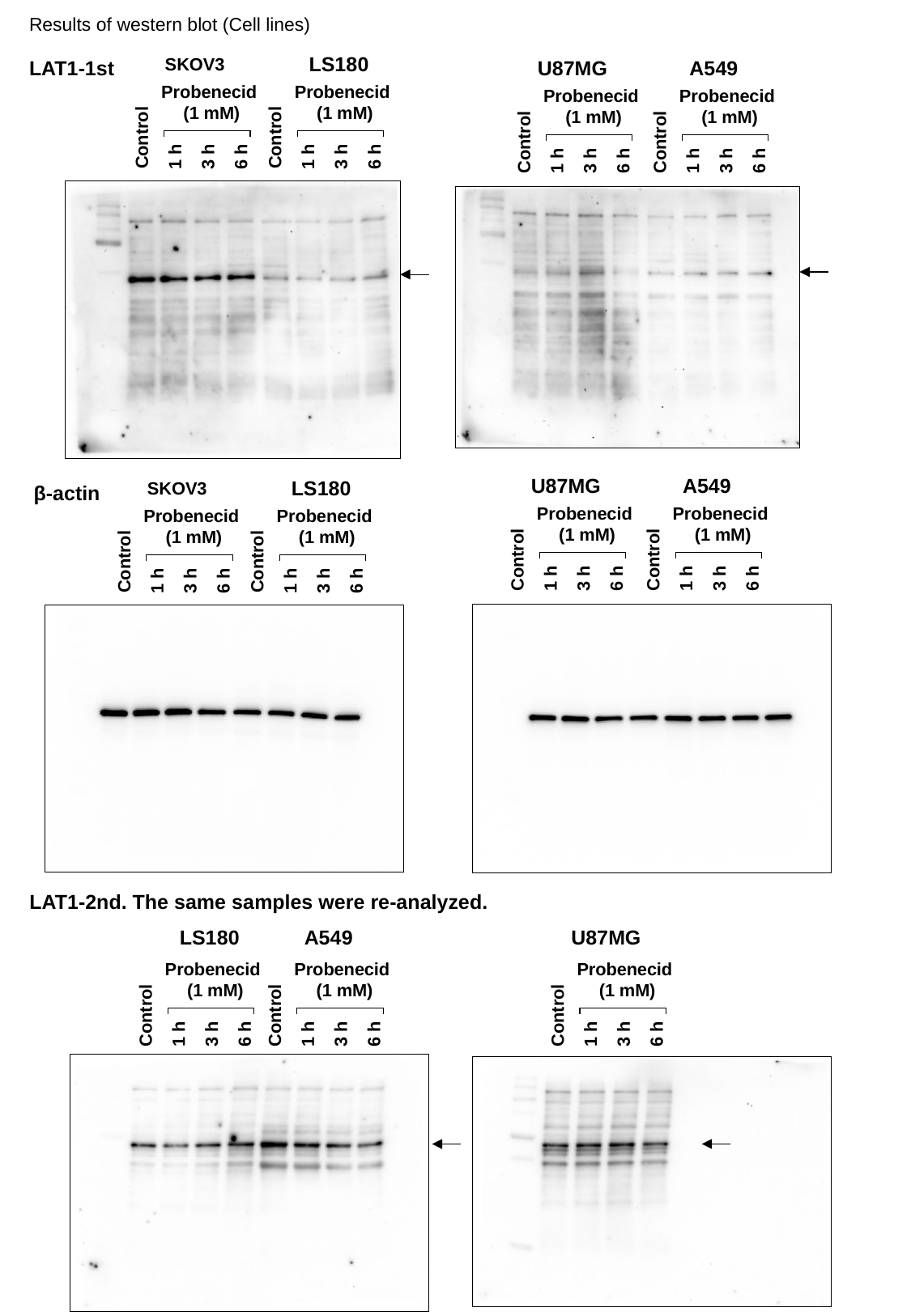

Results of western blot (Cell lines)
LS180
SKOV3
LAT1-1st
U87MG
A549
Probenecid
(1 mM)
Control
1 h
3 h
6 h
Probenecid
(1 mM)
Control
1 h
3 h
6 h
Probenecid
(1 mM)
Control
1 h
3 h
6 h
Probenecid
(1 mM)
Control
1 h
3 h
6 h
U87MG
A549
LS180
SKOV3
β-actin
Probenecid
(1 mM)
Control
1 h
3 h
6 h
Probenecid
(1 mM)
Control
1 h
3 h
6 h
Probenecid
(1 mM)
Control
1 h
3 h
6 h
Probenecid
(1 mM)
Control
1 h
3 h
6 h
LAT1-2nd. The same samples were re-analyzed.
LS180
A549
U87MG
Probenecid
(1 mM)
Control
1 h
3 h
6 h
Probenecid
(1 mM)
Control
1 h
3 h
6 h
Probenecid
(1 mM)
Control
1 h
3 h
6 h

## Slide 3
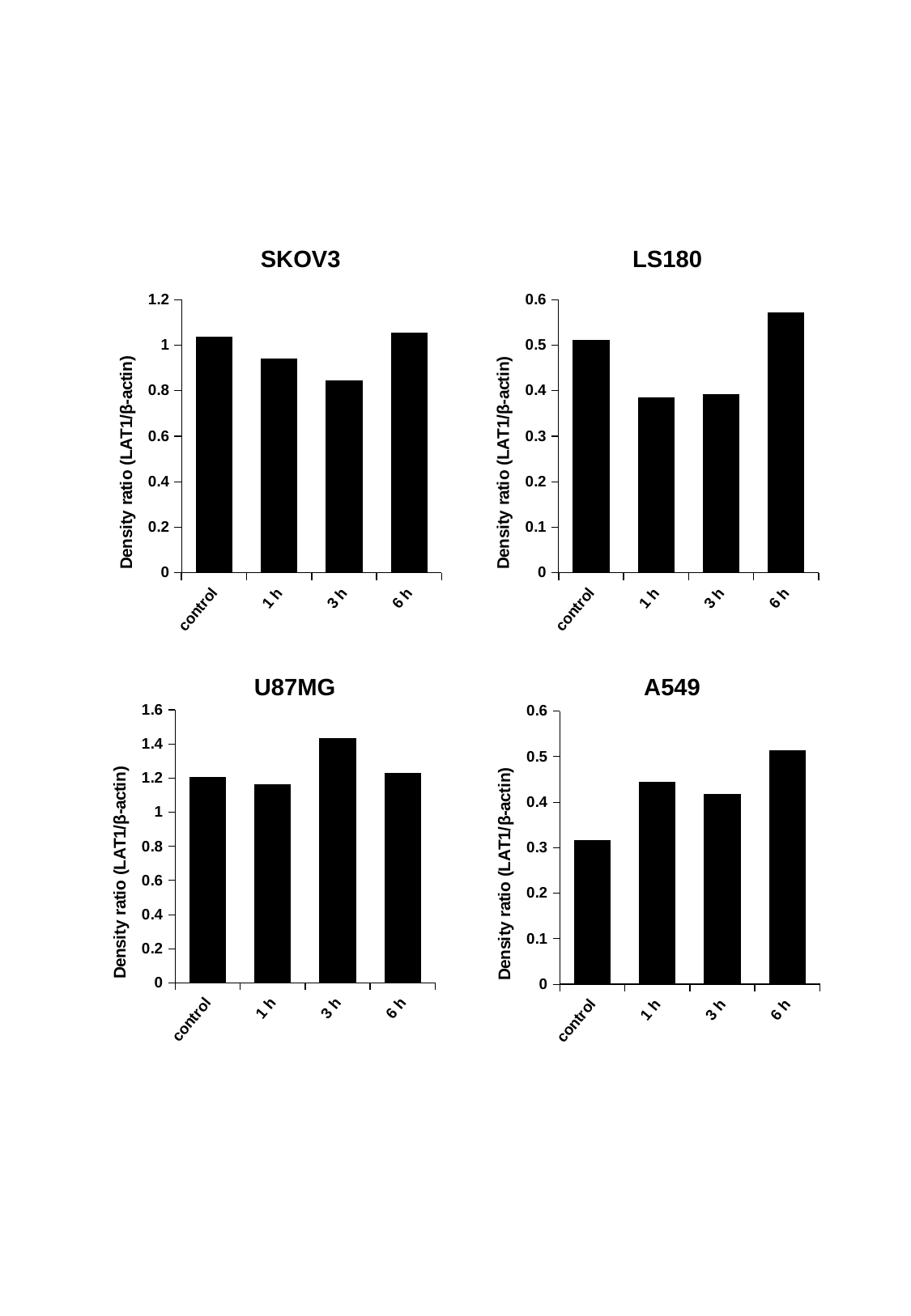

SKOV3
LS180
### Chart
| Category | |
|---|---|
| control | 1.0344957939905162 |
| 1 h | 0.9406385099989928 |
| 3 h | 0.8426523969094798 |
| 6 h | 1.0540429762350914 |
### Chart
| Category | |
|---|---|
| control | 0.5105386106229767 |
| 1 h | 0.3836790100260904 |
| 3 h | 0.3917895259245975 |
| 6 h | 0.5706965537396725 |U87MG
A549
### Chart
| Category | |
|---|---|
| control | 1.2025093989475655 |
| 1 h | 1.1622355573306344 |
| 3 h | 1.4293097759953384 |
| 6 h | 1.2278855548293093 |
### Chart
| Category | |
|---|---|
| control | 0.3150251572680471 |
| 1 h | 0.44307656182466515 |
| 3 h | 0.41733628178136684 |
| 6 h | 0.5132727092602904 |
